# mRNA therapeutics in cancer immunotherapy

**DOI:** 10.1186/s12943-021-01348-0

**Published:** 2021-04-15

**Authors:** Jan D. Beck, Daniel Reidenbach, Nadja Salomon, Ugur Sahin, Özlem Türeci, Mathias Vormehr, Lena M. Kranz

**Affiliations:** 1 BioNTech SE, An der Goldgrube 12, 55131 Mainz, Germany; 2 TRON - Translational Oncology at the University Medical Center of the Johannes Gutenberg-University gGmbH, Freiligrathstraße 12, 55131 Mainz, Germany

**Keywords:** Cancer immunotherapy, Messenger RNA, Cancer vaccines, Antibodies, Immunomodulatory proteins, Immunoreceptors, CARs

## Abstract

Synthetic mRNA provides a template for the synthesis of any given protein, protein fragment or peptide and lends itself to a broad range of pharmaceutical applications, including different modalities of cancer immunotherapy. With the ease of rapid, large scale Good Manufacturing Practice-grade mRNA production, mRNA is ideally poised not only for off-the shelf cancer vaccines but also for personalized neoantigen vaccination. The ability to stimulate pattern recognition receptors and thus an anti-viral type of innate immune response equips mRNA-based vaccines with inherent adjuvanticity. Nucleoside modification and elimination of double-stranded RNA can reduce the immunomodulatory activity of mRNA and increase and prolong protein production. In combination with nanoparticle-based formulations that increase transfection efficiency and facilitate lymphatic system targeting, nucleoside-modified mRNA enables efficient delivery of cytokines, costimulatory receptors, or therapeutic antibodies. Steady but transient production of the encoded bioactive molecule from the mRNA template can improve the pharmacokinetic, pharmacodynamic and safety properties as compared to the respective recombinant proteins. This may be harnessed for applications that benefit from a higher level of expression control, such as chimeric antigen receptor (CAR)-modified adoptive T-cell therapies. This review highlights the advancements in the field of mRNA-based cancer therapeutics, providing insights into key preclinical developments and the evolving clinical landscape.

## Structure and pharmacology of synthetic mRNA

**Fig. 1:**
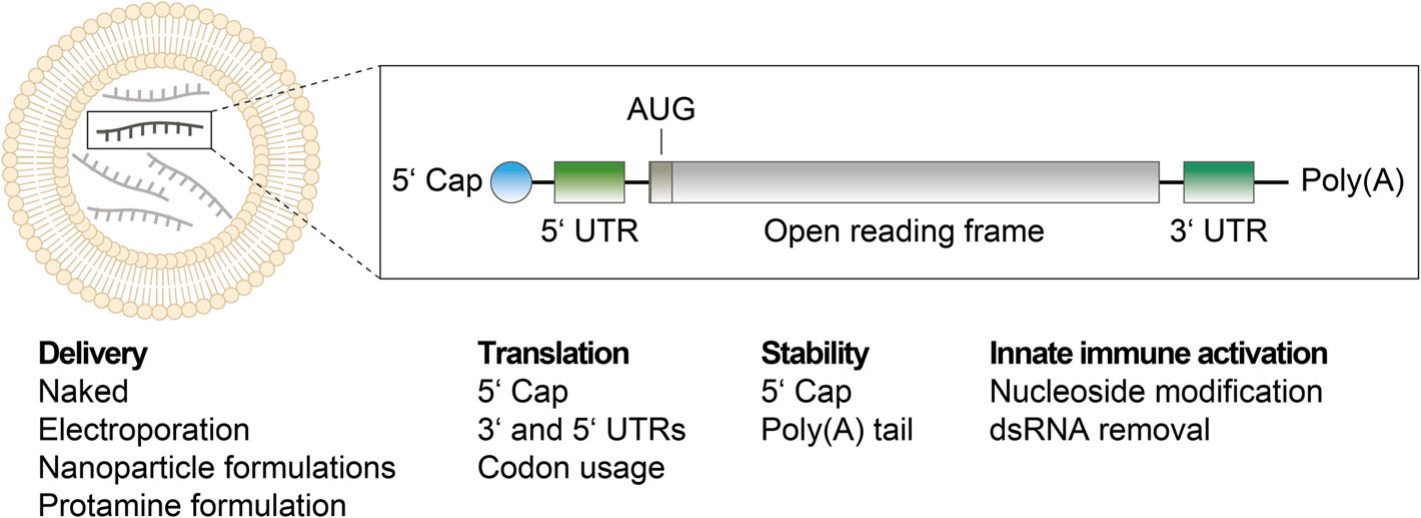
Delivery and structural elements of mRNA therapeutics. Structure of a lipid-based mRNA nanoparticle (left) and synthetic mRNA (right), comprising a 5′ cap, 5′ and 3′ UTRs, a start codon initiating the open reading frame (AUG), and a poly(A) tail. Listed are different mRNA delivery methods, as well as tunable structural elements influencing mRNA translation, stability and potential of innate immune activation. UTR: untranslated region; ds: double-stranded

mRNA was long considered insufficiently stable for pharmaceutical applications, given its susceptibility to rapid degradation by ubiquitous RNases. Over the last 30 years, extensive efforts have been made to increase intracellular stability, translational efficiency and uptake of mRNA. These optimizations were achieved by modification of its non-coding elements (5′ cap structure and its capping efficiency [1–4], 5′- and 3′-untranslated regions (UTRs) [5–9], 3′ poly(A) tail [5, 10, 11]) and of the coding region [12], and through the development of transfection and formulation technologies (Fig. 1).

Building on these advances, synthetic mRNA has emerged as a versatile delivery system for genetic information to induce the production of peptides and proteins by cells.

Synthetic mRNA is single-stranded (ss), contains a 5′ cap, UTRs embracing the coding region and a 3′ poly(A) tail, thus resembling naturally occurring processed mature mRNA molecules, and is generated by in vitro transcription (IVT) from a linear DNA template. Exogenous mRNA enters the cell either by directly passing through the cytoplasmic membrane (e.g., if electroporated), or by endocytosis followed by endosomal escape (if delivered as naked or formulated mRNAs). mRNA does not enter the nucleus, nor integrates into the genome. Translation occurs in the cytosol and the protein derived from the synthetic mRNA is not distinguishable from protein translated from endogenous mRNA. The protein undergoes post-translational modifications and is routed to subcellular compartments, such as the secretory pathway, the cell membrane, the nucleus, mitochondria or peroxisomes, via targeting sequences or transmembrane domains. Eventually, the protein is degraded and peptides are presented on major histocompatibility (MHC) complexes.

In parallel to its translation to protein, exogenous mRNA exerts its activity as a natural ligand of endosomal toll-like receptors (TLRs) 3, 7 and 8, or of retinoic acid-inducible gene 1 (RIG-I) and melanoma differentiation-associated protein 5 (MDA5) in the cytoplasm, and triggers the release of type I interferon (IFN) and pro-inflammatory cytokines (reviewed in Pastor et al. [13]), providing mRNA with strong intrinsic adjuvanticity.

**Fig. 2:**
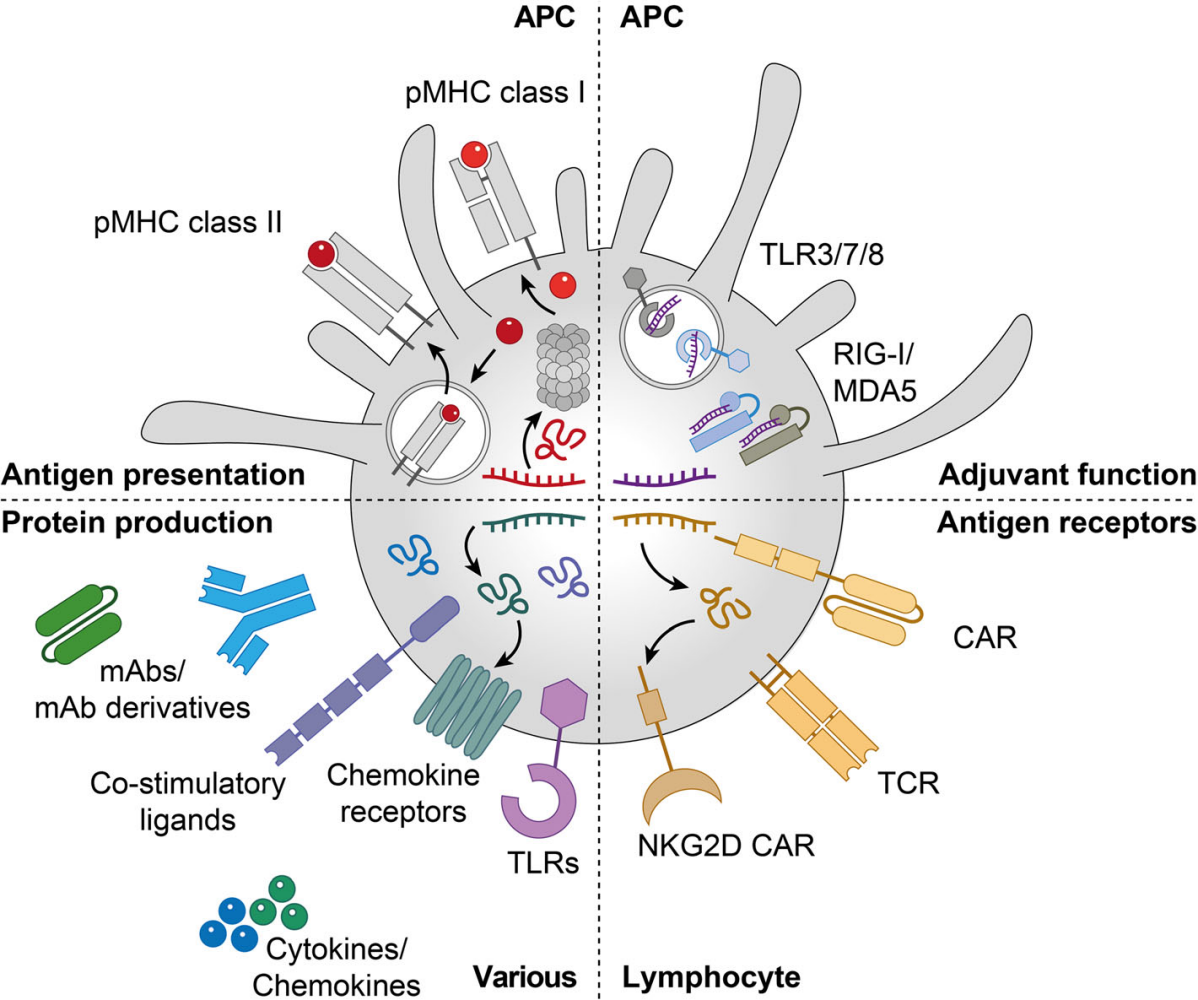
mRNA therapeutics in cancer immunotherapy. mRNA is used for anti-cancer vaccination, where it delivers cancer antigens to APCs for the presentation on MHC class I and II (top left) and stimulates innate immune activation by binding to PRRs expressed by APCs (top right), introduces antigen receptors such as CARs and TCRs into lymphocytes (bottom right), and allows the expression of immunomodulatory proteins including TLRs, chemokine receptors, co-stimulatory ligands, cytokines, chemokines and different mAb formats in various cell subsets (bottom left). APC: antigen-presenting cell; CAR: chimeric antigen receptor; mAb: monoclonal antibody; MDA5: melanoma differentiation-associated protein 5; pMHC: peptide-major histocompatibility complex; NKG2D: natural killer group 2D; PRR: pattern recognition receptors; RIG-I: retinoic acid-inducible gene 1; TCR: T-cell receptor; TLR: Toll-like receptor

In cancer immunotherapy, the most advanced application of mRNA is therapeutic vaccination, which leverages both the capability of mRNA to deliver genetic information and its innate immunostimulatory activity. The latter is particularly important for breaking immune tolerance when cancer-associated self-antigens are targeted. However, IFN-stimulated genes (ISGs), such as IFN-inducible double-stranded (ds)RNA-activated protein kinase (PKR), and 2′,5′-oligoadenylate synthetase (OAS) with subsequent RNase L expression, initiate a state of anti-viral defense, characterized by stalled mRNA translation and increased targeting of mRNA for degradation (reviewed in Kroczynska et al. [14], Munir et al. [15]). Other applications of mRNA in cancer immunotherapy include the engineering of T cells and natural killer (NK) cells with antigen receptors, and its use as a template for immunologically active proteins in a variety of immune and non-immune cells (Fig. 2). Several of these applications rely more heavily on a high area under the curve (AUC) of mRNA translation, and stalled translation is not desirable. Immunostimulatory activity of mRNA can be attenuated by modified nucleosides, such as pseudouridine, N^1^-methylpseudouridine, 2-thiouridine, 5-methylcytidine or N^6^-methyladenosine [16–19], and by the removal of dsRNA [20, 21]. Throughout this review, mRNA nucleoside modifications are explicitly indicated where applicable.

With the global threat of the COVID-19 pandemic accelerating rapid-response vaccine development, mRNA-based therapeutics were shown to deliver on their promise: active at a relatively low dose range, can be developed rapidly, and GMP-compliant manufacturing processes easily upscaled for rapid availability of large numbers of doses [22–24]. It is to be expected that lessons learned in the context of COVID-19 vaccine development can be leveraged to further advance the development of mRNA–based cancer immunotherapies.

## mRNA-based cancer vaccines

Cancer vaccination involves the induction of tumor-specific T-cell responses potentially capable of tumor rejection by providing cancer antigens in the context of immunostimulation. Some cancer vaccines targeting surface exposed antigens additionally aim at inducing a tumor-specific B-cell response. Antigens are either tumor-associated self antigens (TAA), such as differentiation antigens, overexpressed antigens, cancer/testis antigens, or truly tumor-specific antigens (TSAs) not subject to immune tolerance, such as viral and mutated neoantigens. Various formats can be used for antigen delivery, including viral vectors, DNA, peptides, mRNA, or dendritic cells (DCs) pulsed with any of these.

mRNA has emerged as an attractive cancer vaccine format as it provides both antigen delivery and innate immune activation-mediated co-stimulation in a spatiotemporally aligned manner. mRNA vaccines encode the full or partial sequence of a TSA or TAA, and do not rely on prior identification of a patient’s human leukocyte antigen (HLA) haplotype or epitope prediction.

The feasibility of mRNA-based cancer vaccination was first demonstrated about 25 years ago [25, 26]. Since then, numerous preclinical and clinical studies explored mRNA for anti-cancer vaccination, either by loading it ex vivo on autologous DCs for adoptive transfer or by direct injection. A summary of all active, and completed or terminated phase II and III clinical trials using mRNA vaccines is provided in Table 1.

**Table 1:** List of all active, and completed or terminated phase II/III clinical trials of mRNA vaccines in cancer therapy

Sponsor	Vaccine type (delivery route)	Indication	ClinicalTrial.gov identifier (phase, allocation)	Combination	Opened	Status
*DC vaccine*						
Antwerp University Hospital	WT1 mRNA (i.d.)	Myeloid leukemia, multiple myeloma	NCT00965224 (II, randomized)	Standard-of-care	2009	Unknown [27]
		AML	NCT01686334 (II, randomized)	Conventional chemotherapy	2012	Recruiting
		GBM	NCT02649582 (I/II)	Temozolomide, temozolomide-based chemoradiation	2015	Recruiting
		Malignant pleural mesothelioma	NCT02649829 (I/II)	Conventional chemotherapy	2017	Recruiting
Argos Therapeutics	Total tumor mRNA and CD40L mRNA (i.d.)	RCC	NCT00678119 (II, single arm)	Standard-of-care (sunitinib)	2008	Completed [28]
			NCT01582672 (III, randomized)		2012	Terminated (Lack of effiacy) [29, 30]
Asterias Biotherapeutics, Inc.	hTERT mRNA with a LAMP-1 targeting sequence (i.d.)	AML (complete remission)	NCT00510133 (II, single arm)	None	2007	Completed [31]
Baylor College of Medicine	Tumor mRNA and tumor lysate (i.d.)	Pancreatic cancer	NCT04157127 (I)	Adjuvant to chemotherapy	2020	Recruiting
Duke University	LAMP-fused HCMV pp65 mRNA (i.d.) and td	Malignant neoplasms of brain	NCT00639639 (I)	None	2006	Active, not recruiting [32]
			NCT02366728 (II, randomized)	Temozolomide, basiliximab (antagonistic anti-CD25)	2015	Active, not recruiting [33]
		Glioblastoma	NCT03688178 (II, randomized)	Temozolomide, varlilumab (agonistic anti-CD27)	2020	Recruiting
	LAMP-fused HCMV pp65 mRNA, GM-CSF (i.d.) and td	Malignant neoplasms of brain	NCT03615404 (I)	None	2018	Active, not recruiting
		Glioblastoma	NCT03927222 (II, single arm)	Temozolomide	2019	Recruiting
Guangdong 999 Brain Hospital	Tumor mRNA	Brain cancer, neoplasm metastasis	NCT02808416 (I)	None	2016	Active, not recruiting
		Recurrent glioblastoma	NCT02808364 (I)	None	2016	Active, not recruiting
	Glioblastoma TAA mRNA (i.d. and i.v.)	Glioblastoma	NCT02709616 (I)	Conventional chemo- and radiotherapy	2016	Active, not recruiting
Hasumi International Research Foundation	IKKb-matured DCs with tumor mRNA, TAA mRNA (gp100, tyrosinase, PRAME, MAGE-A3, IDO) and neoAg mRNA (GNAQ/GNA11Q209 or R183) (i.v.)	Uveal metastatic melanoma	NCT04335890 (I)	None	2020	Recruiting
Herlev Hospital	PSA, PAP, survivin, hTERT mRNA (i.d.)	Metastatic prostate cancer	NCT01446731 (II, randomized)	Docetaxel	2011	Completed [34]
Immunomic Therapeutics, Inc.	HCMV pp65-shLAMP or pp65-flLAMP, GM-CSF and td (both)	GBM, glioblastoma, malignant glioma, astrocytoma	NCT02465268 (II, randomized)	Temozolomide	2016	Recruiting
Memorial Sloan Kettering Cancer Center	Langerhans-type DCs with Trp2 mRNA	Melanoma	NCT01456104 (I)	None	2011	Active, not recruiting
	Langerhans-type DCs with CT7, MAGE-A3, WT1 mRNA	Multiple myeloma	NCT01995708 (I)	Standard-of-care	2014	Active, not recruiting
Oslo University Hospital	Tumor mRNA and hTERT, survivin TAA mRNA	Prostate cancer	NCT01197625 (I/II)	None	2010	Active, not recruiting
Radboud University	Tyrosinase, gp100 mRNA (i.d. and i.v.)	Melanoma	NCT02285413 (II, randomized)	Cisplatin	2011	Completed [35]
University Hospital Erlangen	Tumor mRNA	Uveal melanoma	NCT01983748 (III, randomized)	None	2014	Recruiting
University of Campinas	WT1 mRNA	Myelodysplastic syndromes, AML	NCT03083054 (I/II)	None	2016	Active, not recruiting
University of Florida	Total tumor mRNA and ex vivo expanded lymphocytes (i.v. and i.d.)	Medulloblastoma, neuroectodermal tumor	NCT01326104 (I, II)	None	2010	Active, not recruiting [36]
	Total tumor mRNA, ex vivo expanded lymphocytes, GM-CSF and td	Diffuse intrinsic pontine glioma, Brain stem glioma	NCT03396575 (I)	Cyclophosphamide + fludarabine lymphodepleting conditioning or temozolomide	2018	Recruiting
Universitair Ziekenhuis Brussel, Bart Nens	MAGE-A3, MAGE-C2, tyrosinase and gp100 mRNA, co-electroporated with TriMix (CD70, CD40L, caTLR4) mRNA	Melanoma	NCT01302496 (II, single arm)	Ipilimumab (antagonistic anti-CTLA-4)	2011	Completed [37–39]
		Melanoma	NCT01676779 (II, randomized)	None	2012	Completed [39]
*Direct injection of mRNA*						
BioNTech SE	RNA-LPX with NY-ESO-1, MAGE-A3, tyrosinase and TPTE (i.v.)	Advanced melanoma	NCT02410733 (I)	None	2015	Active, not recruiting [40, 41]
	RNA-LPX with TNBC TAAs, p53 and neoAgs (warehouse) (i.v.)	TNBC	NCT02316457 (I)	None	2016	Active, not recruiting
	RNA-LPX with ovarian TAAs (i.v.)	Ovarian cancer	NCT04163094 (I)	Carboplatin and paclitaxel	2019	Recruiting
	RNA-LPX with prostate TAAs (i.v.)	Prostate cancer	NCT04382898 (I/II)	Cemiplimab (antagonistic anti-PD-1) and/or goserelin acetate	2019	Recruiting
	RNA-LPX with HPV16 antigens (i.v.)	HPV16^+^ and PD-L1^+^ HNSCC	NCT04534205 (II, randomized)	Pembrolizumab (antagonistic anti-PD-1)	2020	Not yet recruiting
	RNA-LPX with NY-ESO-1, MAGE-A3, tyrosinase and TPTE (i.v.)	Melanoma	NCT04526899 (II, randomized)	Cemiplimab	2020	Not yet recruiting
	RNA-LPX with CLDN6 (i.v.)	Solid tumors	NCT04503278 (I/II)	CLDN6-specific CAR-T cells	2020	Recruiting
Genentech Inc., BioNTech SE	RNA-LPX with tumor neoAgs (i.v.)	Different solid cancers	NCT03289962 (I)	Atezolizumab (antagonistic anti-PD-L1)	2017	Recruiting
		Melanoma	NCT03815058 (II, randomized)	Pembrolizumab	2019	Recruiting
Changhai Hospital; Stemirna Therapeutics	mRNA encoding neoAg (s.c.)	Esophageal squamous carcinoma, gastric-, pancreatic- and colorectal adenocarcinoma	NCT03468244 (NA)	None	2018	Recruiting
CureVac AG	Protamine-formulated mRNA (RNActive) PSA, PSMA, PSCA, STEAP1, PAP and MUC1 (i.d.)	Prostate cancer	NCT01817738 (I/II, randomized)	None	2012	Terminated [42–44]
			NCT02140138 (II, randomized)	Radical prostatectomy	2014	Terminated (after enrollment of 35/36 patients)
eTheRNA	mRNA encoding tyrosinase, gp100, MAGE-A3, MAGE-C2, and PRAME and TriMix (CD40L, CD70 and caTLR4) mRNA (i.n.)	Melanoma	NCT03394937 (I)	None	2018	Recruiting [45]
Ludwig Institute for Cancer Research, CureVac AG, Böhringer Ingelheim	RNActive encoding NY-ESO-1, MAGE-C1, MAGE-C2, TPBG, survivin, MUC1 (i.d.)	Meatastatic NSCLC	NCT03164772 (I/II)	Durvalumab (antagonistic anti-PD-L1), Tremelimumab (antagonistic anti-CTLA-4)	2017	Recruiting
Merck Sharp & Dohme Corp.	LNP-formulated mRNA encoding different KRAS mutations (i.m.)	KRAS-mutant NSCLC, colorectal cancer, pancreatic adenocarcinoma	NCT03948763 (I)	Pembrolizumab	2019	Recruiting
ModernaTX Inc., Merck Sharp & Dohme Corp.	Lipid-formulated mRNA encoding neoAg (i.m.)	Solid tumors	NCT03313778 (I)	Pembrolizumab	2017	Recruiting [46]
		Melanoma	NCT03897881 (II, randomized)	Pembrolizumab	2019	Recruiting
University of Florida	Lipid-formulated mRNA with tumor and LAMP-fused HCMV pp65 TAA mRNA (i.v.)	Adult glioblastoma	NCT04573140 (I)	None	2020	Not yet recruiting

From ClinicalTrial.gov (keywords: cancer, mRNA, vaccine) on Nov 1, 2020 and PubMed literature search. *AML* acute myeloid leukemia, *ca* constitutively active, *CLDN6* claudin-6, *CTLA-4* cytotoxic T-lymphocyte-associated protein 4, *GBM* glioblastoma multiforme of brain, *GM-CSF* granulocyte-macrophage colony stimulating factor, *HCMV* human cytomegalovirus, *HNSCC* head and neck squamous cell carcinoma, *HPV* human papilloma virus, *hTERT* telomerase reverse transcriptase, *i.d.* intradermal, *i.m.* intramuscular, *i.n.* intranodal, *i.v.* intravenous, *IDO* indoleamine-pyrrole 2,3-dioxygenase, *IKKb* inhibitor of nuclear factor kappa-B kinase subunit beta, *LAMP* lysosome-associated membrane glycoprotein, *MAGE* melanoma-associated antigen, *MUC1* mucin-1, *neoAg* neoantigen, *NY-ESO-1* New York esophageal squamous cell carcinoma-1, *NSCLC* non-small-cell lung cancer, *PAP* prostatic acid phosphatase, *PD-1* programmed cell death protein 1, *PD-L1* Programmed cell death ligand 1, *PRAME* melanoma antigen preferentially expressed in tumors, *PSA* prostate-specific antigen, *PSCA* prostate stem cell antigen, *PSMA* prostate-specific membrane antigen, *RCC* renal cell carcinoma, *RNActive* Protamine-formulated mRNA, *RNA-LPX* liposome-formulated mRNA, *s.c.* subcutanous, *STEAP* 6-transmembrane epithelial antigen of prostate, *TAA* tumor-associated antigen, *td* tetanus-diphtheria toxoid, *TLR* toll-like receptor, *TNBC* triple-negative breast cancer, *TPBG* trophoblast glycoprotein, *TPTE* transmembrane phosphatase with tensin homology, *Trp2* tyrosinase-related protein-2, *WT1* Wilm’s tumor protein 1

### mRNA-based DC vaccines

As professional antigen presenting cells (APCs), DCs constantly engulf cellular material from their surroundings. To efficiently deliver tumor antigen to DCs, many early studies focused on ex vivo DC loading and re-infusion of the transfected cells to patients via the subcutaneous, intranodal (injected into a particular lymph node) or intravenous route. The generation of DC vaccines requires isolation of monocytes or hematopoietic progenitor cells from the blood (leukapheresis), ex vivo cultivation, differentiation and tumor-antigen loading and is labor-intensive, costly and complex (detailed review by Perez et al. [47]).

#### Loading DCs with tumor antigens

One of the approaches to pulse DCs with mRNA encoding tumor antigens is the use of bulk mRNA extracted from autologous tumors. Whole tumor mRNA-loaded DC vaccines demonstrated safety in phase I and I/II clinical trials in patients with renal cell carcinoma (RCC; NCT00006431) [48], pediatric and adult brain cancer [49], pediatric neuroblastoma [50], melanoma (NCT01278940) [51], and androgen-resistant prostate cancer [52]. In a phase I/II study (NCT01278940), whole tumor mRNA-transfected DC vaccines were administered alone or together with interleukin (IL)-2 and induced T-cell responses against mRNA-transfected DCs ex vivo in 16 of the 31 patients with advanced melanoma [53]. Immune-responders exhibited significantly enhanced survival in comparison to non-immune-responders (14 vs. 6 months). As the mRNA is extracted from the individual patient’s tumor, feasibility depends on sample availability and quality. Vaccine production is therefore highly variable and sometimes impossible. Within the extracted mRNA, truly cancer-associated antigen encoding mRNAs represent a very small fraction. A low number of relevant epitopes needs to compete with the majority of irrelevant peptides for presentation on MHC molecules for T-cell priming. In vitro mRNA amplification attempting to increase the abundance of all mRNA species in bulk preparations including those representing potential tumor rejection antigens, may even promote under-representation or loss of immunogenic antigens or epitopes in the process [54].

Another approach is to resort to defined TAAs and generate synthetic mRNA for ex vivo DC transfection, as has been done with prostate-specific antigen (PSA) [55], carcinoembryonic antigen (CEA) [56, 57], gp100/tyrosinase [58–60], human telomerase reverse transcriptase (hTERT) [61] and Wilms’ tumor 1 (WT1) antigen [27, 62]. TAA mRNA DC vaccines exhibited good safety profiles in phase I and I/II clinical trials without serious adverse effects, such as autoimmunity, and did not show dose-limiting toxicity. In a phase II clinical study (NCT00965224), patients with acute myeloid leukemia (AML) in remission were vaccinated with WT1 mRNA-electroporated DCs [27]. Prevention or delay of relapse was observed in 43% of patients and the five-year overall survival of the vaccinated patients compared favorably to historic controls.

In a randomized phase II clinical trial (NCT01446731), a DC vaccine with mRNA encoding PSA, prostatic acid phosphatase (PAP), survivin and hTERT failed to significantly increase the disease-specific survival of docetaxel-treated, castration-resistant prostate cancer patients (docetaxel: 21.9 months, docetaxel and DC vaccination: 25.1 months) [34]. TAA-specific T-cell responses were present in 9 of the 18 combination therapy-treated patients, of which three were de novo.

In 78 melanoma patients with resected regional lymph node metastases (NCT00940004, NCT01530698, NCT02285413, NCT00243529), vaccination with gp100- and tyrosinase-mRNA loaded DC vaccines as an adjuvant treatment showed a favorable safety profile and induced tumor-specific T-cell responses in 71% of the patients [63]. The overall survival of DC-vaccinated patients was doubled as compared to that of 209 matched controls (63.6 months vs. 31.0 months).

Generally speaking, T-cell responses induced by mRNA-loaded DC vaccines have been rather weak, which may contribute to the low clinical efficacy. DC differentiation, maturation and antigen loading directly impact DC homing and T cell co-stimulation (reviewed in Gu et al. [64]), and are targets for further improvement of this approach.

#### Engineering DC antigen presentation

Cognate T-cell help is a critical factor that determines the extent of vaccine-induced T-cell responses. This requires the vaccine antigen to be presented on MHC class II molecules of the DC. mRNA transfected into DCs undergoes cytoplasmic translation, enters the endogenous processing compartment and is therefore presented on MHC class I rather than MHC class II molecules. Several strategies have been followed to enhance MHC class II antigen presentation by routing the antigen through lysosomal compartments, such as fusion of the vaccine antigen to lysosome-associated membrane proteins (LAMPs) [65, 66], or to the MHC class I cytoplasmic and transmembrane domain [67]. Fusion of a lysosomal targeting signal (LAMP-1) to CEA in a chimeric mRNA (CEA/LAMP-1) enhanced the expansion of CEA-specific CD4^+^ T-cell responses and led to a more potent CEA-specific CTL response in vitro [68]. CEA LAMP mRNA-transfected DC vaccines were evaluated in phase I studies in patients with newly diagnosed glioblastoma multiforme (NCT02529072, NCT00626483, NCT00639639) [32, 33, 69, 70], which suggested prolongation of overall survival as compared to non-transfected DC vaccines or historical controls [33]. Four ongoing phase II clinical trials are evaluating CEA/LAMP-1 mRNA engineered DC vaccines in glioblastoma patients in combination with tetanus toxoid preconditioning to enhance DC migration, granulocyte-macrophage colony-stimulating factor (GM-CSF), and other drugs, such as basiliximab (antagonistic anti-CD25 monoclonal antibody (mAb)), varilumab (agonistic anti-CD27 mAb) or temozolomide (NCT02366728, NCT03927222, NCT03688178, NCT02465268).

#### Co-stimulatory ligands and receptors

As ssRNA and dsRNA trigger TLR activation, pulsing of DCs with mRNA provides a maturation signal irrespective of the biological activity of the encoded antigen. Several strategies aim to further improve the T-cell priming capacity of tumor-antigen loaded DC vaccines. These include co-transfection with immunostimulatory ligands and receptors for enhanced DC maturation and T-cell co-stimulation, which can be introduced by mRNA. In preclinical studies, DC co-transfection of cancer antigens with OX40 ligand (OX40L), 4-1BB ligand (4-1BBL), glucocorticoid-induced tumor necrosis factor receptor (GITR) ligand (GITRL), CD40 ligand (CD40L), constitutively active (ca)CD40 or inducible T cell co-stimulatory ligand (ICOSL) mRNA improved DC co-stimulation and resulted in enhanced T-cell priming [71–76]. One of the most clinically advanced DC vaccines is Rocapuldencel-T, an autologous tumor mRNA-transfected DC vaccine co-transfected with CD40L mRNA [28, 29]. In a phase III trial with 462 metastatic RCC (mRCC) patients (NCT01582672), Rocapuldencel-T failed to improve the overall survival of sunitinib-treated mRCC patients. Co-transfecting TAA mRNA-loaded DC vaccines with CD40L, CD70 and caTLR4 mRNA (TriMix) represents an alternative approach [77–79]. Superior T-cell priming capacity was observed by T-cell stimulation with TriMix-transfected DCs compared to that induced by irrelevant mRNA-transfected immature DCs, or by DCs matured in culture supplemented with cytokines (IL-1β, IL-6, tumor necrosis factor (TNF)α, and prostaglandin E2 (PGE2)) [77]. In a single-arm phase II clinical study, TriMix-DCs, transfected with TriMix and four TAAs (melanoma-associated antigen (MAGE)-A3, MAGE-C2, tyrosinase, or gp100) fused to an MHC class II targeting signal, plus the mAb ipilimumab (blocking the immune checkpoint cytotoxic T-lymphocyte-associated protein 4 (CTLA-4)) were combined in 39 pretreated advanced melanoma patients (NCT01302496) [37]. The combination was well tolerated and after a median follow-up of 36 months, eight complete and seven partial responses were observed. T-cell responses were detected in 12 of 15 patients by IFNγ enzyme-linked immunospot (ELISpot) [38]. The study lacked a direct comparison of TriMix-DCs and ipilimumab against ipilimumab alone.

#### Co-stimulatory cytokines

mRNA-encoded cytokines are another class of molecules used to enhance DC maturation and T-cell priming capacity. In the context of autologous DC vaccines loaded with whole tumor mRNA preparations or with synthetic TAA-encoding mRNA, co-transfection with cytokine-encoding mRNA, such as GM-CSF, IL-12 and IL-15, was explored [80–84]. Co-transfection of GM-CSF mRNA into tumor mRNA-loaded DCs significantly enhanced the anti-tumor efficacy of DC vaccines in CT26 tumor-bearing mice, which was associated with increased cytotoxicity of bulk splenocytes against CT26 tumor cells in vitro [80]. In another study, co-delivery of IL-12 mRNA enhanced the ex vivo migratory and immunostimulatory capacity of healthy donor-derived human DCs transfected with melanoma antigen recognized by T cells (MART)-1 [81]. Proliferation, effector function and memory potential of MART-1-specific CD8^+^ T cells were significantly enhanced by co-culture with IL-12 and MART-1 mRNA-transfected DCs compared to MART-1 mRNA-transfected DCs alone.

### mRNA-based direct cancer vaccines

The direct injection of mRNA is an alternative to DC vaccines and circumvents the necessity of DC isolation, ex vivo cultivation and re-infusion. Injected mRNA is taken up by local cells, including APCs, and translocates to the cytoplasm for translation. Various mRNA vaccines are currently being evaluated, using different nanoparticle formulations, delivery routes and structural backbones, of which the most advanced candidates are summarized below.

#### Non-formulated mRNA vaccines

Non-formulated or “naked” mRNA administered intradermally or into lymph nodes (intranodally) has been shown to prime T-cell responses in mice [85–88] and humans [45, 89, 90]. The rationale for intranodal injection is to deliver the vaccine directly into the area of T-cell priming for uptake by resident DCs. The major uptake mechanism of naked mRNA by immature DCs in lymph nodes [91] and in the dermis [92] is macropinocytosis. The feasibility and safety of naked mRNA vaccines was first demonstrated in a phase I/II trial in 15 metastatic melanoma patients using intradermally injected autologous tumor mRNA and GM-CSF [93]. In five of 13 evaluable patients, enhanced antigen-specific CD4^+^ and CD8^+^ T-cell responses in ex vivo expanded PBMC were detected by intracellular IFNγ staining upon re-stimulation with tumor mRNA-transfected PBMC. Vaccine-induced humoral responses were analyzed by incubating allogenic melanoma cell lines with plasma samples taken before or during the therapy followed by flow cytometry analysis to detect cell-bound IgG antibodies. Upon vaccination, an increase in the amount of tumor-cell specific IgG antibodies was observed in the plasma of four out of the 13 patients. Objective responses were not apparent in this trial. In a phase I/II clinical study with 30 stage IV RCC patients, intradermal administration of naked mRNA encoding mucin 1 (MUC1), CEA, human epidermal growth factor receptor-2 (HER-2)/neu, telomerase, survivin and MAGE-A1 combined with GM-CSF was well tolerated and associated with stable disease in 15 patients and one partial response [90]. Antigen-specific T-cell responses were observed by IFNγ ELISpot or chromium-release cytotoxicity assays in 12 out of 17 patients.

In 2012, a phase I study of 29 advanced melanoma patients was initiated, which proved that intranodal injection of mRNA is both feasible and safe (NCT01684241) (unpublished results). Vaccination induced either de novo or expanded existing T-cell responses against the vaccine-encoded antigens New York esophageal squamous cell carcinoma-1 (NY-ESO-1) and tyrosinase in six out of 13 evaluable patients. New lesions occurred in only eight of the 29 patients during the course of the study. In another phase I study of 20 melanoma patients (NCT03394937), intranodal injection of tyrosinase, gp100, MAGE-A3, MAGE-C2, and melanoma antigen preferentially expressed in tumors (PRAME)-encoding mRNA together with TriMix mRNA was well tolerated. As observed by IFNy ELISpot and intracellular cytokine staining, T-cell responses against at least one TAA were induced in 35% of patients [45].

Mutated neoantigens are promising vaccine targets as they are abundant in many tumor types and highly tumor-specific (reviewed in Vormehr et al. [94]). Neoantigen-specific T cells are likely not subject to central tolerance and have a higher likelihood to induce high-affinity T-cell responses, while TAAs come with the challenge of breaking tolerance. However, as each single tumor has its unique profile of mutations, the full potential of mutated neoantigens as an ideal source for vaccine antigens can only be leveraged in the context of individualized vaccines, with each and every patient being administered their personal set of mutated epitopes. As mRNA vaccines can be produced fast and cost-effectively, they are particularly suited for the purpose of “on demand” vaccine design and manufacturing. Intranodal administration of a neoantigen-specific mRNA vaccine, comprising 20 patient-specific mutations, was investigated in a phase I clinical trial in stage III and IV melanoma patients (NCT02035956) [89]. Neoantigen-specific mRNA vaccines induced CD4^+^ and CD8^+^ T-cell responses against multiple vaccine neoepitopes in 13 evaluable melanoma patients. Objective responses were observed and the cumulative rate of recurrences was significantly reduced by vaccination, resulting in a sustained progression-free survival. The vaccine was well tolerated.

#### Formulated mRNA vaccines

Nanoparticulate formulations protect mRNA from extracellular RNases and improve uptake by APCs in vivo. Formulations typically contain polymers, such as protamine, or cationic and ionizable lipids with or without polyethylene glycol (PEG) derivatives to enable complexing with mRNA via electrostatic interactions and condensing of the RNA molecules (reviewed in Zeng et al. [95]).

One of the mRNA formulations in advanced clinical development is based on protamine, a polycationic peptide that complexes negatively charged mRNA for intradermal delivery [42–44, 96–100]. In mice, protamine-formulated mRNA is taken up by leukocytes and non-leukocytes in the skin, including APCs that migrate to the draining lymph node, express and present the encoded protein and induce T-cell responses [96]. The safety and feasibility of a protamine-formulated mRNA vaccine encoding MART-1, tyrosinase, gp100, MAGE-A1, MAGE-A3 and survivin was demonstrated in 21 metastatic melanoma patients (NCT00204607) [99]. The mRNA vaccine was injected intradermally with GM-CSF either alone or in conjunction with keyhole limpet hemocyanin as adjuvants [101]. Antigen-specific T cell responses in ex vivo expanded PBMC were detected by intracellular IFNγ staining after re-stimulation with irradiated, autologous PBMC transfected with TAA mRNA. Vaccination was associated with an increase in the number of antigen-specific CD4^+^ and CD8^+^ T cells in two of four immunologically evaluable patients. One complete response occurred in seven patients with measurable disease. In addition, a vaccine encoding six protamine-formulated prostate cancer-specific antigens (PSA, prostate-specific membrane antigen (PSMA), prostate stem cell antigen (PSCA), 6-transmembrane epithelial antigen of prostate (STEAP), PAP and MUC1) was investigated in a phase I/II trial in patients with asymptomatic or minimally symptomatic metastatic castrate-resistant prostate cancer (NCT01817738) [42–44]. The vaccine was well tolerated. In the phase I part, vaccine-induced antigen-specific T-cell responses were shown in 26 of 33 patients by ex vivo IFNγ ELISpot assay, intracellular cytokine and tetramer flow cytometry [44]. In the randomized, placebo-controlled phase II part of the study (*n* = 197) [43], the vaccine failed to improve overall survival and progression-free survival. A vaccine derived from the same protamine-formulated mRNA platform encoding five tumor-associated antigens (NY-ESO-1, MAGE-C1, MAGE-C2, survivin and trophoblast glycoprotein (TPBG)) was further evaluated in a phase I/IIa dose-escalation trial in seven patients with locally advanced and 39 patients with metastatic non-small-cell lung cancer (NSCLC) and controlled disease after first-line therapy (NCT00923312) [100]. The vaccine was well-tolerated and induced T-cell responses against one or more antigens in 63% of evaluable patients. The median overall survival, however, was not improved by vaccine monotherapy compared to that typically seen with maintenance chemotherapy. An ongoing phase I/II trial is currently evaluating this vaccine, with the addition of the antigen MUC1 in combination with either the antagonistic anti-programmed death-ligand 1 (PD-L1) antibody durvalumab or durvalumab plus the antagonistic anti-CTLA-4 antibody tremelimumab in patients with metastatic NSCLC (NCT03164772).

A major limitation of local administration is that only the injected or the draining lymph nodes are accessed and participate in antigen-specific T-cell priming. Other formulations are required for intravenous administration to deliver mRNA to secondary lymphoid organs body-wide, including the spleen as the largest agglomeration of immune cells. Formulation technologies developed for intravenous administration not only protect RNA integrity, but also promote its targeted expression in lymph node-resident DCs.

mRNA lipoplexes (RNA-LPX) are based on cationic liposomes as broadly used for transfection reagents in vitro [102]. Specific delivery of nanoparticles to APCs and improved uptake for presentation of epitopes derived from the encoded antigens can be achieved by decorating such particles with targeting moieties, such as antibodies, single chain variable fragments (scFv) fragments and small molecules binding DEC-205, mannose receptors and C-type lectin domain family 9 (CLEC9A) (reviewerd in Phuha et al. [103]; [104, 105]). Alternatively, RNA-LPX can be modified to have a negative net charge when prepared by molar excess of negatively charged mRNA, or by shielding positive surface charges with PEG. mRNA-LPX with a negative net charge are subject to specific uptake by APCs in vivo without the need for targeting moieties (reviewed in Guan et al. [106]).

Intravenously administered negatively charged to near-neutral RNA-LPX vaccines have been shown in mice to target APCs resident in lymphoid organs, including the spleen, lymph nodes and bone marrow, while inducing strong local (cell-specific activation) and systemic (proinflammatory cytokine abundance in the circulation) type I IFN-dominated immune modulation [40]. In several mouse tumor models, RNA-LPX vaccines induce strong effector and memory T-cell responses directed against the encoded TSAs or TAAs, and mediate tumor growth control or rejection, and improve survival [40, 107–109]. In a phase I clinical trial (NCT02410733), 119 melanoma patients were vaccinated with an RNA-LPX vaccine encoding the four melanoma TAAs MAGE-A3, transmembrane phosphatase with tensin homology (TPTE), NY-ESO-1 and tyrosinase [40, 41]. In the subset of antagonistic anti-programmed cell death protein-1 (PD-1) antibody-experienced patients with evaluable disease at baseline (*n* = 25), vaccination alone mediated one metabolic complete response, three partial responses and seven cases of stable disease. In combination with anti-PD-1 antibodies (pembrolizumab or nivolumab, *n* = 17), six partial responses and two cases of stable disease were observed. RNA-LPX vaccine-induced CD4^+^ and CD8^+^ T-cell responses were observed in the majority of patients reaching up to low double-digit percentages of total circulating CD8^+^ T cells. A randomized phase II study will investigate this melanoma TAA-specific RNA-LPX vaccine in combination with the antagonistic anti-PD-1 antibody cemiplimab (NCT04526899). The same platform encoding other tumor type specific TAAs is being evaluated in various phase I and I/II clinical trials in patients with triple negative breast cancer (TNBC; NCT02316457), ovarian cancer (NCT04163094) and prostate cancer (NCT04382898).

Moreover, several clinical trials are testing this intravenous RNA-LPX technology in conjunction with TSAs, which are expected to be less compromised by central immune tolerance. One example is a human papilloma virus (HPV)16 E6 and E7 mRNA-encoding RNA-LPX vaccine, which is being evaluated in a randomized phase II trial in combination with pembrolizumab in patients with HPV16^+^ and PD-L1^+^ head and neck squamous cell carcinoma (HNSCC) (NCT04534205). Another example is the so-called individualized neoantigen-specific immunotherapy (iNeST) program, which treats each patient with their ‘on-demand’ manufactured individualized set of cancer mutations. This truly personalized treatment approach is being tested in various tumor types and treatment lines in the phase I and II studies NCT03289962, NCT03815058, and NCT04486378.

The same RNA-LPX vaccine platform was utilized to expand chimeric antigen receptor (CAR)-engineered T cells against claudin 6 (CLDN6), a carcinoembryonic cell surface antigen, in mice [110]. Vaccination with CLDN6-encoding RNA-LPX resulted in the expression of CLDN6 on splenic DCs and macrophages in vivo and increased the proportion of proliferating CLDN6-CAR-T cells after adoptive transfer. Subtherapeutic doses of CLDN6-CAR-T cells were expanded by the CLDN6 RNA-LPX vaccine, resulting in regression of subcutaneous CLDN6^+^ Lewis lung tumors. Safety and efficacy of this concept are currently being evaluated in a phase I/II clinical study in patients with solid cancers (NCT04503278).

Lipid nanoparticles (LNPs) are mRNA formulations based on a mixture of cholesterol, ionizable and helper lipids, and PEG-derivatives [106]. Intramuscularly administered RNA-LNPs have recently gained attention, as this is the format of the first two SARS-CoV-2 vaccines to be approved, based on their demonstration of compelling efficacy in COVID-19 protection [22, 111–117], which was in line with previous preclinical and clinical data showing induction of strong antigen-specific immune responses against other infectious diseases [118–120]. Both BNT162b2 and mRNA-1273 contain N^1^-methylpseudouridine-modified mRNA with blunted innate immune modulatory activity to promote potent T_H_1, T follicular helper and germinal center B cell responses for the induction of neutralizing antibodies as part of the multi-effector immune response [115, 121, 122].

In the cancer therapeutic field, intramuscularly administered, LNP-formulated nucleoside-modified mRNA vaccines encoding 20 patient-specific mutated neoepitopes are currently being evaluated in combination with pembrolizumab in patients with solid cancer (NCT03313778, phase I) and melanoma (NCT03897881, randomized phase II). Data from a recent phase I clinical trial in four gastrointestinal cancer patients (NCT03480152) suggest that vaccination with neoantigen-specific RNA-LNP is safe and induces mutation-specific T-cell responses [123] with no objective clinical responses observed yet in the limited number of patients tested.

Besides the clinically explored mRNA formulations discussed above, a plethora of different in vivo DC-targeting mRNA nanoparticles are under development. A recent study analyzed over 1000 mRNA lipid formulations for transfection efficacy. A subset of these were tested for in vivo protein production, APC maturation in local lymph nodes, induction of T and B cell responses in naïve, and anti-tumor efficacy of Trp2, OVA and E7-encoding mRNA lipid formulations in B16, B16-OVA and TC-1 tumor-bearing mice [124]. The formulations performing best shared a common structure involving an unsaturated lipid tail, a dihydroimidazole linker and a cyclic amine head group and mediate APC maturation by signaling through the STING pathway. Insights such as these will promote rational approaches for formulation development.

## mRNA-encoded antigen receptors

Another immunotherapy approach in cancer is based on the redirection of T cells against tumor cells by stable integration of tumor-specific antigen receptors, either with T cell receptors (TCRs) which recognize MHC-presented epitopes from both intra- and extracellular antigens, or CARs which bind to tumor surface antigens and are MHC-independent. T cells are then expanded in cell culture systems. Patients are treated with a lymphodepleting chemotherapy regimen prior to adoptive cell transfer (ACT) of such engineered cell products to improve engraftment.

In the ACT field, CD19-directed CAR-T cells are the most advanced. Several products have been market authorized for the treatment of B-cell leukemia and lymphoma [125, 126] based on response rates of over 80%, half of which were complete responses. Usually retro- or lentiviral technologies are applied to engineer antigen-receptor expressing cells. Stably transduced CAR-T cells are highly potent and are living drugs, the efficacy of which is associated with their capability to persist. When developing CAR-T cells against new antigens that are not yet vetted clinically for target safety, transient expression of an mRNA-encoded CAR may be risk-mitigating. Also, in contrast to viral transduction, mRNA does not pose the risk of genomic integration of the transgene and malignant transformation. mRNA transfection further allows the co-delivery of additional mRNAs for concomitant manipulation of lymphocytes, such as immunostimulatory cytokines [127], chemokine receptors or adhesion molecules for targeted migration [128–131], or Cas9-mediated genome editing [132].

### Generating CAR-T cells with mRNA

Electroporation of mRNA CARs is a practical and scalable method accomplishing lymphocyte transfection rates of more than 90% without affecting the viability of the cell product, and is used in the vast majority of studies [133–136]. mRNA electroporation results in CAR expression on the surface of T cells for around 7 days [137–140] and CARs internalized upon target cell encounter are not restored [139, 141–144]. The molecular structure of the CAR construct (e.g., co-stimulatory domains) may impact the level and duration of expression [145–148]. While the level and persistence of CAR expression correlate positively with potency of killing, high CAR expression was shown in vitro to favor activation-induced cell death (AICD) [138], and high amounts of mRNA input can affect viability [149]. Optimization of structural elements including the 5′ cap, UTRs and poly(A) tail [150, 151] improves CAR expression levels and duration, and may circumvent the need for high mRNA input amounts. In addition, dsRNA removal and N^1^-methylpseudouridine modification have been shown to enhance CAR expression and result in more functional CAR-T cells [152], but have not yet been applied in clinical mRNA CAR studies.

mRNA CAR-T cells must be administered repeatedly, in contrast to classical CAR-T cells which constitutively express the CAR. Consequently, mRNA CAR-T cells are produced in large quantities and cryopreserved in batches that can be thawed for each treatment cycle. GMP-compliant protocols for the large-scale manufacturing of mRNA CAR-T cells have been established [153, 154], and freezing mRNA CAR-T cells was demonstrated not to negatively affect their function [136, 138, 145, 148]. For the treatment of solid tumors, intratumoral injection is a way to prevent T cells from losing CAR expression before reaching the tumor cells [143]. Clinical development programs need to implement proper follow-up of CAR T cell persistence kinetics to develop treatment schedules and define dosing intervals, which may differ depending on the preparation of the engineered cell product.

### Preclinical and clinical testing of mRNA-encoded CARs

Early studies with TCRs specific for NY-ESO-1, MART-1 or p53 [133] and a CAR directed against CD19 [155] showed that mRNA-transfected T cells efficiently lyse antigen-expressing tumor cells. First evidence for anti-tumor effects in vivo came from PBLs transfected with mRNA encoding a HER-2/neu-specific CAR in an ovarian cancer xenograft model [134]. Clinical phase I studies explored mRNA CAR-T cells directed against various antigens (Table 2). Treatment with mRNA CAR-T cells targeting CD19 was shown to be well tolerated in pilot phase I studies in pediatric (NCT02624258) and adult (NCT02277522) patients with classical Hodgkin lymphoma. Patients not only underwent lymphodepleting chemotherapy prior to CAR-T cell infusion, but also in between CAR-T cell cycles to deplete T cells that had lost CAR expression, using a lymphodepletion protocol that had enhanced the efficacy of repeated mRNA CAR-T cell delivery in preclinical models [162]. Two treatment cycles were conducted, each representing three CAR-T cell infusions 2 to 3 days apart with lymphodepletion 1 or 4 days before each cycle. One patient of four evaluable patients experienced a complete response. Assessment of CAR mRNA transcripts in the blood of this patient 2 days after each adoptive transfer confirmed the presence of the infused cells. In the remaining patients, no CAR-T cells were detected in the majority of samples taken 2 days after infusion [163].

**Table 2:** List of all active, completed or terminated clinical trials of mRNA CAR-T cells in cancer therapy

Sponsor	Therapy	Indication	**ClinicalTrial.gov** identifier (phase)	Combination	Opened	Status
**Cartesian Therapeutics**	BCMA CAR-T cells (Descartes-08)	Multiple myeloma	NCT03448978 (I)	Fluodarabine and cyclophosphamide pretreatment	2018	Recruiting [156]
**The Third Affiliated Hospital of Guangzhou Medical University**	NKG2D-ligand targeting CAR-NK cells	Solid tumors	NCT03415100 (I)	IL-2	2018	Unknown [157]
**University of Pennsylvania**	Mesothelin CAR-T cells	Malignant pleural mesothelioma	NCT01355965 (I)	None	2011	Completed [158, 159]
		Metastatic PDAC	NCT01897415 (I)	None	2013	Completed [159, 160]
	c-MET CAR-T cells	Metastatic breast cancer, TNBC	NCT01837602 (I)	None	2013	Completed [161]
		Melanoma, breast cancer	NCT03060356 (I)	None	2016	Terminated (lack of funding)
	CD19 CAR-T cells	Hodgkin lymphoma	NCT02277522 (I)	Interval cyclophosphamide [162]	2014	Terminated (inability to meet enrollment goal) [163]
			NCT02624258 (I)	Interval cyclophosphamide [162]	2015	Terminated (PI’s decision) [163]
	CD123 CAR-T cells	Relapsed or refractory AML	NCT02623582 (I)	Cyclophosphamide pretreatment	2015	Terminated (lack of funding) [164]

From ClinicalTrial.gov (keywords: cancer, mRNA, CAR) on Nov 1, 2020 and PubMed literature research. *AML* acute myeloid leukemia, *BMCA* B cell maturation antigen, *CAR* chimeric antigen receptor; *cMET* protein kinase Met; *IL* interleukin, *NK* natural killer, *NKG2D* Natural killer group 2D, *PDAC* pancreatic ductal adenocarcinoma; protein kinase Met, *TNBC* triple-negative breast cancer

Two phase I studies (NCT01355965, NCT01897415) were initiated based on the observation that repeated injection of mesothelin-directed [165] mRNA CAR-T cells prevented tumor outgrowth in solid and disseminated mesothelioma xenograft models [150]. Preliminary analyses of eight out of 34 patients showed that this approach is well tolerated, with one exception: One patient experienced anaphylactic shock, most likely resulting from the induction of IgE antibodies against murine sequences in the CAR construct [158]. One patient with advanced malignant pleural mesothelioma experienced a partial response after three infusions and a second patient with metastatic pancreatic cancer had stable disease and a 40% decrease of tumor cells in ascites after receiving eight intravenous infusions followed by two intratumoral injections [159]. CAR transcripts were detectable in the blood of the patients for up to 3 days after infusion. CAR-T cells persisted longer in ascites and the primary tumor of the pancreatic cancer patient, where transcripts were detectable 6 days after intravenous or 13 days after intratumoral injection, respectively. In six other individuals with pancreatic cancer, stable disease occurred in two patients, and a third patient experienced a partial metabolic response of the target lesion [160].

In AML, lineage markers used as target antigens are expressed by hematopoietic stem cells and myeloid progenitor cells [140, 166]. Targeting antigens such as CD33 or CD123 may cause hematologic toxicity, which is less manageable than the B-cell depletion induced by targeting of CD19. Two studies showed eradication of AML xenografts assessed by CD33 [140] or CD123 [167] mRNA CAR T cells in preclinical mouse models. The self-inactivation of CD123 CAR expression allowed the subsequent recovery of the myeloid compartment by bone marrow transplantation. This approach proceeded to clinical testing in relapsed or refractory AML (NCT02623582). However, in the seven patients who received up to six infusions of CD123 mRNA CAR-T cells, no clinical benefit was observed [164].

mRNA CAR-T cells targeting protein kinase Met (c-Met) are in clinical development for the treatment of breast cancer and melanoma (NCT01837602, NCT03060356) [161], and B-cell maturation antigen (BCMA)-specific mRNA CAR-T cells are being developed as a treatment option for multiple myeloma (NCT03448978) [156].

mRNA CARs are also used for retargeting of γδ T cells [168, 169], NKT cells [170] and NK cells [135, 171, 172]. Besides the classical CAR construct containing an scFv as a targeting moiety, CARs featuring the extracellular domain of natural killer group 2D (NKG2D) have been engineered to recognize tumor cells that express high levels of NKG2D ligands. This approach showed promising results in preclinical studies [157]. In a clinical phase I study using CAR-NK cells (NCT03415100), preliminary signals of clinical activity were observed in three evaluated colorectal cancer patients: two experienced reduction of tumor cells in ascites and stable disease, a third patient had a complete metabolic response of a liver lesion [157].

## mRNA-encoded antibodies

mRNA is also being explored for delivery of full IgG mAbs or scaffolds, including engineered bispecific antibody fragments, and addresses pharmacokinetic (e.g., short half-life, fast clearance) as well as manufacturing challenges (e.g., aggregates) to accelerate clinical development.

mAbs are an established treatment modality in cancer and are used for targeting of tumor cells and for the modulation of immune cell responses. Most of the approved recombinant mAbs are administered intravenously to achieve systemic exposure.

Established standard-of-care mAbs are obvious starting points to explore mAb-encoding mRNA in conjunction with liver-targeting formulations for high protein production in liver-resident cells and release of the protein into the circulation. Rituximab, targeting CD20, was the first mAb approved for cancer therapy and is used for the treatment of non-Hodgkin’s lymphoma and chronic lymphocytic leukemia (CLL) [173]*.* HER-2/neu-targeting mAb trastuzumab is approved for the treatment of HER-2/neu-overexpressing breast cancer and metastatic gastric or gastroesophageal junction adenocarcinoma [174]. In mice bearing human lymphoma xenografts, intravenously administered, LNP-formulated mRNA encoding rituximab induced a more pronounced anti-tumor effect compared to the recombinant protein counterpart [175]. Trastuzumab encoded on mRNA intravenously delivered with a liver-targeting LNP formulation and recovered from mouse serum exerted antibody-dependent cellular cytotoxicity (ADCC). The treatment prolonged survival in a HER-2/neu-positive breast cancer xenograft model [176].

Engineered bispecific mAbs, comprising an anti-CD3 scFv fused to a tumor antigen-specific scFv, are able to redirect T cells to tumor cells and promote tumor cell killing [177]. The small molecular weight of bispecific mAbs allows efficient penetration of tumor tissue but comes with the drawback of a short serum half-life. mRNA with reduced immunostimulatory activity was investigated for the systemic delivery of bispecific mAbs targeting CD3 and either CLDN6, CLDN18.2 or epithelial cell adhesion molecule (EpCAM) [178]. Polymer-lipid-based transfection reagent-formulated, N^1^-methylpseudouridine-modified and dsRNA-purified bispecific mAb-encoding mRNA (called RiboMAB) was translated in the liver after intravenous administration and resulted in sustained plasma antibody concentration over several days in mice. In contrast, non-nucleotide modified and non-dsRNA purified mRNA induced only minimal plasma concentrations of bispecific mAbs, highlighting the importance of engineering mRNA for enhanced protein production. The favorable pharmacokinetic profile of modified and purified mRNA was associated with the induction of T-cell cytotoxicity and superior anti-tumor activity against human ovarian cancer xenografts as compared to recombinant mAb.

In contrast to systemic exposure mediated through intravenous injection, local mRNA translation by direct injection into a specific tissue or by transfection of specific cell types ex vivo restricts the therapeutic activity of the translated protein to the target cells or their intermediate surroundings. Intratumoral injection of mouse tumors with an antagonistic anti-PD-L1 mAb-encoding self-amplifying (sa)RNA, which is derived from alpha- or flaviviruses and encodes both the protein of interest and a replicase for RNA amplification, resulted in superior anti-tumor efficacy compared to the intraperitoneally or intratumorally administered recombinant protein counterpart. Intratumoral saRNA administration mediated anti-PD-L1 mAb translation in transfected cells and promoted CD8^+^ T cell infiltration into the tumor [179].

Adoptive transfer of DCs transfected ex vivo with mRNA encoding an agonistic anti-GITR mAb delayed tumor growth in mice in the absence of an antigen-specific immunization, with efficacy being comparable to that of intravenous delivery of high amounts (1 mg) of recombinant anti-GITR mAb [180]. The administration of DCs co-transfected with mRNAs encoding an agonistic anti-GITR mAb or a soluble GITRL-Fc fusion protein, antagonistic anti-CTLA-4 mAb and Trp-2 mRNA further expanded this approach. The combined transfection of GITRL-Fc, anti-CTLA-4 mAb and Trp-2 mRNA led to significant prolonged survival of mice compared to either Trp-2 mRNA or Trp-2 and anti-CTLA-4 mAb mRNA or Trp-2 and GITRL-Fc mRNA-transfected DCs [181]. In vitro, these multi-transfected DCs improved the cytotoxicity of CTLs while inhibiting the induction of regulatory CD4^+^ T cells (Tregs). The concept was subsequently explored in a small trial including two patients with metastatic melanoma (NCT01216436) (Table 3).

**Table 3:** List of all active, completed or terminated clinical trials of mRNA antibodies and immunomodulators in cancer therapy

Sponsor	Therapy (delivery route)	Indication	ClinicalTrial.gov identifier (phase, allocation)	Combination	Opened	Status
*Antibodies*						
Duke University	DC with GITRL/anti-CTLA-4 mAb mRNA (i.n.)	Metastatic melanoma	NCT01216436 (I)	Melanoma TAA mRNA transfected DCs	2010	Terminated (lack of personnel)
*Immunomodulators*						
*Cytokines*						
Medimmune LLC, Moderna TX Inc.	LNP with IL-12 mRNA (i.t.)	Solid tumors	NCT03946800 (I)	Durvalumab	2019	Recruiting
Moderna TX Inc., AstraZeneca	LNP with OX40L, IL-23 and IL-36γ mRNA (i.t.)	Relapsed/refractory solid tumor malignancies or lymphoma, TNBC, HNSCC, Non-Hodgkin lymphoma, urothelial cancer	NCT03739931 (I)	Durvalumab	2018	Recruiting [182]
Sanofi, BioNTech SE	scIL-12, IL-15sushi, IFNα and GM-CSF mRNA (i.t.)	Metastatic neoplasms	NCT03871348 (II, non-randomized)	Cemiplimab	2019	Recruiting
*Stimulatory ligands and receptors*						
eTheRNA Immunotherapies Nv.	TriMix (CD70, CD40L, caTLR4) mRNA and TAA mRNA (i.n.)	Melanoma	NCT03394937 (I)	None	2018	Recruiting
Moderna TX Inc.	LNP-OX40L mRNA (i.t.)	Relapsed/refractory solid tumor malignancies or lymphoma, ovarian cancer	NCT03323398 (I/II, non-randomized)	Durvalumab	2017	Recruiting
Universitair Ziekenhuis Brussel, eTheRNA Immunotherapies Nv.	TriMix (CD70, CD40L, caTLR4) mRNA (i.t.)	Early resectable breast cancer	NCT03788083 (I)	None	2018	Recruiting

From ClinicalTrial.gov (keywords: cancer AND mRNA AND immunomodulator OR cytokine OR co-stimulator OR antibody) on Dec 1, 2020 and PubMed literature search. *ca* constitutively active, *CD40L* CD40 ligand, *CTLA-4* cytotoxic T-lymphocyte-associated protein 4, *DC* dendritic cell,* GITRL* glucocorticoid-induced tumor necrosis factor receptor family-related protein ligand, *GM-CSF* granulocyte-macrophage colony stimulating factor, *HNSCC* head and neck squamous cell carcinoma, *i.n.* intranodal, *i.t.* intratumoral, *IFN* interferon, *IL* interleukin, *LNP* lipid nanoparticle, *mAb* monoclonal antibody, *OX40L* OX40 ligand, *sc* single-chain, *TAA* tumor-associated antigen, *TLR* toll-like receptor, *TNBC* triple-negative breast cancer

To increase bispecific mAb concentration and persistence in the tumor proximity, T cells were transfected with mRNA encoding the CD19-targeting bispecific T cell enganger (BiTE) blinatumomab approved for the treatment of certain types of acute lymphoblastic leukemia (ALL). Secretion of the BiTE led to the release of inflammatory cytokines and efficient killing of tumor cells by transfected T cells in vitro [183]. These observations correlated with enhanced in vivo anti-tumor efficacy upon infusion of mRNA-electroporated T cells in a mouse model of leukemia.

## mRNA-encoded immunomodulators

mRNA-encoded immunomodulators investigated so far comprise cytokines as well as co-stimulatory ligands and receptors (see Table 3 for those in clinical development).

When using mRNA to produce immunomodulators in vivo, a high protein yield is desirable. The majority of studies that relied on mRNA translation upon intravenous or intratumoral administration used nucleoside-modified mRNA, while approaches involving ex vivo transfected target cells did not attempt to attenuate the inherent immunostimulatory activity of the mRNA.

### Cytokines

Recombinant T cell-stimulating cytokines are a preclinically validated treatment concept [184–186], the clinical development of which is hampered by substantial toxicity.

IL-2 is a key cytokine involved in differentiation, proliferation, survival and effector function of T cells and has been approved for the treatment of melanoma and RCC [187]. Major limitations of recombinant IL-2 are its preferential stimulation of Treg expansion and its short serum half-life, demanding high and frequent dosing which in turn potentiates adverse effects. The rationale for using mRNA to express IL-2 in vivo is to prolong the production of the cytokine, reducing peak serum concentration in favor of protracted activity. Two IL-2 encoding investigational nucleoside-modified RNA-LNP products are in development. One encodes an IL-2 with an extended half-life and is developed in combination with an IL-7 encoding RNA-LNP [188, 189]. In mice, systemic administration of these two cytokine RNA-LNPs synergistically enhanced vaccine-mediated induction of antigen-specific CD8^+^ T cells and increased the ratio of antigen-specific CD8^+^ T cells over Tregs, translating into superior control of syngeneic tumors. The second RNA-LNP codes for an engineered IL-2 variant with reduced Treg bias [188, 189]. The IL-2 variant was generated by rational design with the objective of decreasing the binding affinity to the IL-2 receptor (IL-2R) α chain primarily expressed by Tregs, and increasing the binding affinity to the IL-2Rβ chain expressed by all T cells, including effector T cells. In syngeneic tumor-bearing mice, intravenous administration of the IL-2 variant RNA-LNP expanded spontaneous anti-tumor T-cell responses without significant elevation of Tregs, leading to a strong increase of the CD8^+^ T cell to Treg ratio. The IL-2 variant RNA-LNP proved effective in syngeneic mouse tumor models, and the anti-tumor effect was significantly enhanced in combination with RNA-LPX vaccination or PD-1/PD-L1 checkpoint inhibition.

IL-15 is functionally related to IL-2, as both signal via IL-2Rβ and the common γ chain (IL-2Rβγ), and thereby stimulate T cells and NK cells [190]. Whereas IL-2 is crucial for the development and homeostasis of Tregs and supports AICD to control auto-reactive T cells, IL-15 prolongs the survival of CD8^+^ memory T cells [191]. IL-15 has a short serum half-life and cancer patients that received intravenous infusion of recombinant IL-15 experienced dose-limiting toxicity [192]. Signaling is induced when IL-15 presented on membrane-associated IL-15Rα binds IL-2Rβγ receptor complexes on T cells in *trans*. To avoid systemic exposure to IL-15, CD8^+^ T cells were transfected ex vivo with mRNA encoding an IL-15-IL-15Rα fusion construct (IL-15sushi). Autonomous stimulation of the trimeric IL-15R in *cis* enhanced the cytotoxicity of the T cells in vitro and when administered to mice, these cells proliferated better [193].

IL-12 is a potent mediator of T_H_1 immunity and exerts significant anti-tumor activity in mouse tumor models. In humans, IL-12 is associated with potentially lethal toxicity upon systemic administration [194, 195]. One study investigated T cells transfected with mRNA encoding a single-chain IL-12 (scIL-12) consisting of the p35 and p40 subunits. Intratumoral injection of scIL-12-expressing T cells resulted in complete rejection of both injected and distant tumor lesions in syngeneic and xenograft mouse models. Co-electroporation of T cells with 4-1BBL mRNA further increased anti-tumor efficacy [196]. Local expression of IL-12 was also achieved through intratumoral delivery of LNPs containing scIL-12 N^1^-methylpseudouridine-modified mRNA. In mouse tumor models, a single intratumoral injection promoted tumor regression, which required IFNγ and CD8^+^ T cells [197]. In a corresponding phase I clinical trial, the human scIL-12 MEDI1191 is being evaluated in patients with solid tumors (NCT03946800). In a different approach, N^1^-methylpseudouridine-modified scIL-12 RNA-LNPs administered intravenously to mice bearing hepatocellular carcinomas reduced liver tumor burden and prolonged survival, without apparent liver toxicity [198]. Reporter mRNA-containing LNPs demonstrated that the translated protein was confined to the tumors and non-malignant regions of the liver and the spleen.

Various mRNAs can be easily mixed and formulated together as a single drug product, a technological property that has encouraged the development of combinatorial approaches. Intratumoral administration of LNP-formulated N^1^-methylpseudouridine-modified mRNAs encoding IL-36γ (a pro-inflammatory cytokine acting as alarmin), IL-23 (a regulator of inflammation) and OX40L demonstrated regression of established tumors in three syngeneic tumor models [199]. Inflammatory cytokines and chemokines in the tumor microenvironment (TME) and recruitment and activation of multiple DC and T cell types increased upon triplet mRNA treatment. These cytokine responses were reproduced in human cell culture studies. A phase I dose escalation study is evaluating the mixture alone or in combination with durvalumab in 126 patients with relapsed/refractory solid tumors or lymphoma (NCT03739931) [182]. Dose expansion cohorts contain patients with TNBC, HNSCC, non-Hodgkin lymphoma, and urothelial cancer. In addition, a mix of scIL-12, IL-15sushi, GM-CSF and IFNα mRNA alone or in combination with cemiplimab is being tested in a phase I clinical trial in 231 patients with metastatic neoplasms (NCT03871348) [200].

In a combination approach using a single mRNA construct, IFNβ was fused with the ectodomain of the transforming growth factor (TGF) β receptor II to stimulate innate immunity while antagonizing the suppressive function of TGFβ [201]. In vitro, the fusion construct increased surface expression of MHC class I and PD-L1 by tumor cells, improved T-cell priming by DCs, and diminished the suppressive capacity of myeloid-derived suppressor cells. This pharmacological profile resulted in tumor growth delay in syngeneic tumor models.

### Stimulatory ligands and receptors

Interactions of stimulatory ligands and receptors provide inflammatory signals to the immune system that can be exploited for cancer immunotherapy. mRNA can be used to transiently equip cells with stimulatory receptors, enabling transient activation of strong inflammatory signals.

Modification of intracellular receptor domains to generate ca receptors is helpful to provide cells with autonomous stimuli. The transfection of T cells, isolated either from PBLs or tumor-infiltrating lymphocytes (TILs), with caTLR4 mRNA, caCD40 mRNA, or both, enhanced the production of IFNγ and TNFα, upregulated 4-1BB and CD25, and increased the cytolytic activity against autologous melanoma cells in vitro [202, 203]. Building on this strategy, the authors of these studies added cytokine mRNAs coding for membrane-anchored variants of IL-2, scIL-12 and IL-15. The membrane-associated cytokines bound their corresponding surface receptors mainly in *cis.* Co-transfection of cytokine mRNAs with caTLR4 mRNA, caCD40 mRNA, or both, enhanced IFNγ production by T cells, induced upregulation of T cell activation molecules (CD25, 4-1BB, OX40), and improved the cytotoxicity of TILs against autologous melanoma cells in vitro in comparison to cells transfected with caTLR4 mRNA, caCD40 mRNA, or both [204, 205].

The transient nature of mRNA translation is appealing for the direct in vivo delivery of stimulatory ligand and receptor-coding mRNA. The injection of naked TriMix mRNA (caTLR4 and co-stimulatory ligands CD70 and CD40L; as described above) directly into the tumor resulted in systemic therapeutic anti-tumor activity in various mouse models and led to a phase I clinical trial in patients with early, resectable breast cancer lesions (NCT03788083) [206]. In addition, LNP-formulated OX40L mRNA entered clinical phase I development in patients with relapsed/refractory solid tumors, lymphoma and ovarian cancer, alone or in combination with durvalumab (NCT03323398). So far, OX40L monotherapy proved to be safe while exerting pro-inflammatory activity in the injected tumor lesions [207].

Resorting to the combination of co-stimulatory ligands and receptors (CD70, OX40L, CD80, CD86), as well as cytokines (IL-12, IFNγ), different mixtures of formulated 5′-methylcytidine- and pseudouridine-modified mRNAs were injected directly into mouse tumors. Among all tested combinations, the triplet of OX40L, CD80 and CD86 mRNA induced the strongest anti-tumor immunity in subcutaneous A20 and CT26 tumor-bearing mice [208].

## Conclusion

The development and worldwide approval or authorization of mRNA vaccines against SARS-CoV-2 within less than 1 year upon occurrence of the COVID-19 pandemic showcased the enormous potential of mRNA technology. COVID-19 mRNA vaccines were rapidly developed, have a favorable safety profile, and outperform established technologies with efficacies around 95% of preventing COVID-19 [24, 114].

This rapid response to the pandemic leveraged the rich source of scientific, clinical, manufacturing and regulatory lessons learned over decades of exploring mRNA technology for the design and clinical development of therapeutic cancer vaccines. In turn, the insights gained in the process of advancing mRNA for the first time through all stages of pharmaceutical drug development to a commercial product will now feed back into its application for cancer immunotherapy.

The beauty of mRNA technology is the broad bandwidth of its versatility. By modifying building blocks, structural elements, and formulations of the synthetic mRNA, a variety of features including targeting to defined cells, duration of expression, and immunological effects can be adapted. This expands the design space for mRNA beyond therapeutic cancer vaccination. mRNA is now utilized for the most diverse immunotherapeutic approaches, including the equipping of immune cells with antigen receptors and the in vivo production of therapeutic proteins such as antibodies or immunomodulators. mRNA CAR-T cells have the potential to improve the safety profile of CAR-T cell therapy and allow concurrent modification of the lymphocytes by co-delivery of additional mRNAs. mRNA-based immunomodulators successfully entered clinical testing. Intratumoral injection of mRNA-encoded cytokines and co-stimulatory ligands demonstrated feasibility and induction of anti-tumor immune responses.

mRNA is expected to become one of the major pillars of drug development. With an increasing number of concepts entering clinical trials, the first approval of an mRNA therapeutic against cancer is nearing.

## Data Availability

Not applicable.

## Abbreviations

ACT: Adoptive cell transfer
ADCC: Antibody-dependent cellular cytotoxicity
AICD: Activation-induced cell death
ALL: Acute lymphoblastic leukemia
AML: Acute myeloid leukemia
APC: Antigen-presenting cell
AUC: Area under the curve
BCMA: B-cell maturation antigen
BiTE: Bispecific T cell engager
BMCA: B cell maturation antigen
ca: Constitutively active
CAR: Chimeric antigen receptor
CD40L: CD40 ligand
CEA: Carcinoembryonic antigen
CLDN6: Claudin 6
CLEC9A: Mannose receptors and C-type lectin domain family 9
CLL: Chronic lymphocytic leukemia
c-Met: Protein kinase Met
CTLA-4: Cytotoxic T-lymphocyte-associated protein 4
DC: Dendritic cell
DcR3: Decoy receptor 3
ds: Double-stranded
EpCAM: Epithelial cell adhesion molecule
ELISpot: Enzyme-linked immune absorbent spot
GITR: Glucocorticoid-induced tumor necrosis factor receptor
GITRL: Glucocorticoid-induced tumor necrosis factor receptor ligand
GM-CSF: Granulocyte-macrophage colony-stimulating factor
HCMV: Human cytomegalovirus
HER-2: Human epidermal growth factor receptor 2
HLA: Human leukocyte antigen
HNSCC: Head and neck squamous cell carcinoma
HPV: Human papilloma virus
hTERT: Human telomerase reverse transcriptase
Ig: Immunoglobulin
i.d.: Intradermal
i.n.: Intranodal
i.t.: Intratumoral
i.v.: Intravenous
ICOSL: Inducible T cell co-stimulatory ligand
IFN: Interferon
IKKb: Inhibitor of nuclear factor kappa-B kinase subunit beta
IL: Interleukin
IL-2R: IL-2 receptor
iNeST: Individualized neoantigen-specific immunotherapy
ISG: IFN-stimulated gene
IVT: In vitro transcription
LAMP: Lysosome-associated membrane protein
MHC: Major histocompatibility complex
LNP: Lipid nanoparticle
mAb: Monoclonal antibody
MAGE: Melanoma-associated antigen
MART: Melanoma antigen recognized by T cells
MDA5: Melanoma differentiation-associated protein 5
mRCC: Metastatic renal cell carcinoma
mRNA: Messenger RNA
MUC1: Mucin 1
neoAg: Neoantigen
NK: Natural killer
NKG2D: Natural killer group 2D
NSCLC: Non-small-cell lung cancer
NY-ESO-1: New York esophageal squamous cell carcinoma-1
OAS: 2′,5′-oligoadenylate synthetase
OX40L: OX40 ligand
PAP: Prostatic acid phosphatase
PD-1: Programmed cell death protein 1
PDAC: Pancreatic ductal adenocarcinoma
PEG: Polyethylene glycol
PGE2: Prostaglandin E2
PKR: IFN-inducible double-stranded RNA-activated protein kinase
PD-L1: Programmed cell death ligand 1
PRAME: Melanoma antigen preferentially expressed in tumors
PRR: Pattern recognition receptors
PSA: Prostate-specific antigen
PSCA: Prostate stem cell antigen
PSMA: Prostate-specific membrane antigen
RCC: Renal cell carcinoma
RIG-I: Retinoic acid-inducible gene 1
RNA-LPX: Liposome-formulated mRNA
sa: Self-amplifying
sc: Single chain
scFv: Single-chain variable fragment
ss: Single-stranded
STEAP: 6-transmembrane epithelial antigen of prostate
STING: Stimulator of interferon gene
TAA: Tumor-associated antigen
TCR: T cell receptor
td: Tetanus-diphtheria toxoid
TGF: Transforming growth factor
TIL: Tumor-infiltrating lymphocytes
TLR: Toll-like receptor
TME: Tumor microenvironment
TNBC: Triple-negative breast cancer
TNF: Tumor necrosis factor
TPBG: Trophoblast glycoprotein
TPTE: Transmembrane phosphatase with tensin homology
Treg: Regulatory CD4^+^ T cell
TriMix: CD40L, CD70 and constitutively active TLR4 mRNA
Trp2: Tyrosinase-related protein-2
TSA: Tumor-specific antigen
UTR: Untranslated region
WT1: WILMS’ tumor 1
4-1BBL: 4-1BB ligand
